# Global transcriptomic responses of *Escherichia coli* K-12 to volatile organic compounds

**DOI:** 10.1038/srep19899

**Published:** 2016-01-28

**Authors:** Pui Yi Yung, Letizia Lo Grasso, Abeed Fatima Mohidin, Enzo Acerbi, Jamie Hinks, Thomas Seviour, Enrico Marsili, Federico M. Lauro

**Affiliations:** 1 Singapore Centre for Environmental Life Sciences Engineering (SCELSE). 60 Nanyang Drive, SBS-01N-27, Singapore 637551; 2 School of Chemical and Biomedical Engineering, Nanyang Technological University, 62 Nanyang Drive, Singapore 637459; 3 School of Biotechnology, Dublin City University, Collins Avenue, Dublin 9, Ireland; 4 Asian School of the Environment, Nanyang Technological University, 50 Nanyang Avenue, N2-01C-45, Singapore 639798

## Abstract

Volatile organic compounds (VOCs) are commonly used as solvents in various industrial settings. Many of them present a challenge to receiving environments, due to their toxicity and low bioavailability for degradation. Microorganisms are capable of sensing and responding to their surroundings and this makes them ideal detectors for toxic compounds. This study investigates the global transcriptomic responses of *Escherichia coli* K-12 to selected VOCs at sub-toxic levels. Cells grown in the presence of VOCs were harvested during exponential growth, followed by whole transcriptome shotgun sequencing (RNAseq). The analysis of the data revealed both shared and unique genetic responses compared to cells without exposure to VOCs. Results suggest that various functional gene categories, for example, those relating to Fe/S cluster biogenesis, oxidative stress responses and transport proteins, are responsive to selected VOCs in *E. coli.* The differential expression (DE) of genes was validated using GFP-promoter fusion assays. A variety of genes were differentially expressed even at non-inhibitory concentrations and when the cells are at their balanced-growth. Some of these genes belong to generic stress response and others could be specific to VOCs. Such candidate genes and their regulatory elements could be used as the basis for designing biosensors for selected VOCs.

Volatile organic compounds (VOCs) are low molecular weight molecules with a vapor pressure of ≥10 Pa at 20 °C^1^, while compounds with a 6-months volatility between 5 and 95% at ambient temperature can be termed semi VOCs (sVOCs)^2^. VOC such as toluene, methyl acetate, trichloroethylene, benzene, and phenol etc., are common indoor and urban contaminants^3^. Examples of common sVOCs include high molecular weight alkanes, polycyclic aromatic hydrocarbons (PAH), organochlorine pesticides, and substitute benzenes^4^,^5^. While there are natural VOCs (e.g. cyclopentanone and dimethyl disulfide) and sVOCs (e.g., n-Heptadecane and 1-butyl-3-methyl- imidazolium hexafluorophosphate) produced biologically during degradation products of plant components or for biochemical signaling^6^,^7^, many VOCs and sVOCs originate from fossil fuels, industrial chemicals and solvents. These compounds present a challenge to receiving environments and wastewater treatment processes, due to their toxicity and low bioavailability for degradation^8^.

The toxicity of VOCs and sVOCs has been evaluated in selected animal models. For example, cyclopentanone, N-methyl-2-pyrrolidone (NMP) and dimethylacetamide (DMA), were found to cause developmental toxicity in rat embryos^9^ and rabbits^10^,^11^. The toxicity of sVOCs commonly found in indoor environment, such as plasticizers, solvents, and flame retardant is also well studied^12^. In industrial settings, VOC and sVOCs have been shown to concentrate in both liquid and gas phases of wastewater treatment plant^13^,^14^. Thus, industrial VOC and sVOC discharges present serious concerns for wastewater treatment.

Microorganisms are constantly sensing and responding to surrounding environmental conditions, including the presence of biologically toxic compounds. VOCs have been found to affect microbial diversity and biodegradation performance in activated sludge^15^ and in soil^16^. Microbial tolerance to various VOCs in bacteria falls into three broad mechanisms: 1) alteration of membrane protein composition^17^,^18^,^19^, 2) export of toxic compounds through membrane transporters^20^,^21^; and 3) to a lesser extent, biotransformation of the compound to less toxic variant, which has been reported for soil microorganisms and a number of *Pseudomonas* species^22^. Expression of detoxifying enzymes such as reductive dehalogenases^23^ and oxygenases^24^, have been exploited in the bioremediation of chlorinated aliphatic hydrocarbons and polycyclic aromatic contaminated soil and groundwater.

The *E. coli* K-12 MG1655 strain used in this study is the primary experimental reference model with a highly curated genome sequence with annotation^25^. It is widely considered the *E. coli* strain of choice and its genome was the first published sequence of a wild-type laboratory strain of *E. coli* K-12 because it has relatively few genetic modifications compared to most other *E. coli* strains. *E. coli* has also been used extensively as biosensor due to its ease of genetic manipulation and availability of information^26^. We chose *E. coli* K-12 also because the GFP: fusion library is readily available^27^. Various genetic mechanisms have been identified to contribute to VOC tolerance in *E. coli*. For example, membrane transport proteins like the acrAB-tolC pump^28^, mannose transporter^29^ and phosphate transporters^30^ in *E. coli* were found to confer tolerance to various VOCs. Regulatory elements such as the FadR, MarR^31^ and purR regulon^32^, were found to be involved in conferring tolerance to n-hexane, p-xylene and cyclohexane. Genes under the central metabolic processes, such as the *cyo* and *nuo* operons, responsible for energy conservation and production, and those under galactitol metabolic process (*gat* genes) were up-regulated in response to ethanol^30^ and butanol^33^, respectively. Overexpression of heat shock proteins, such as the GrpE and GroESL chaperone system also resulted in increased tolerance of various forms of butanol as well as ethanol^34^,^35^. In addition, studies has been conducted to look at tolerance of *E. coli* to butanol using genomic library screening^36^, microarray, and at proteomic, regulatory network and metabolite levels^33^,^37^,^38^.

In most of these studies, genetic responses to sub-toxic VOC and sVOC concentrations have not been described. Studying gene activation/inactivation following exposures to sub-toxic levels will enable mechanisms of adaptation and enhanced tolerance to be decoupled from general stress responses, which would be expected at higher concentrations. In addition, information on the genetic responses of microorganisms to non-inhibitory levels would be relevant to understand and improve VOC and sVOC resistance in microorganisms that can be used for biocatalysis (e.g. for the removal of VOCs and sVOCs) applications. Such information would be preliminary to the development of rapid biosensing of VOC and sVOC in contaminated wastewater, offering protective measures for wastewater treatment plants and final users of reclaimed water^39^.

In this study, we used transcriptomics to investigate the global gene expression of *E. coli* K-12 grown in the presence of industrially relevant VOCs and sVOCs. All of the selected compounds are commonly used as solvents or produced as by-products during manufacturing of polymers, cleaners and industrial chemicals, with an exception of N-methylsuccinimide (NMS), which is one of the metabolites commonly used as a biomarker for exposure of the solvent N-methyl-2-pyrrolidone (NMP)^9^. We aim to understand the specific and non-specific responses to the selected compounds. The focus in this study is to investigate genes that are responsive at non-growth inhibitory concentration, yet significant enough to induce a response at the transcriptome level.

## Results and Discussion

### Growth and overall transcriptome profile of *E. coli* grown with VOCs

We analyzed the transcriptome of *E. coli* K-12 grown in the presence and absence of selected VOCs (Supplementary Figure S1) using Illumina RNA-seq. Growth curve experiments were performed on *E. coli* with 0 (as control), 0.02, 0.1 and 0.5% (v/v) of the selected VOCs to determine the highest non-inhibitory concentration to be used in RNAseq experiments (Supplementary Methods and Figure S2). The concentrations were established to be: 0.02% for toluene (T), 0.1% for n-butanol (B), N-cyclohexyl-pyrrolidone (CHP), cyclopentanone (CP), dimethyl sulfide (DMS), N-methyl-2-pyrrolidone (NMP); 0.5% for N,N – Dimethylacetamide (DMA) and N-methyl succinimide (NMS) (Table 1). At these concentrations the cells reach optical density (600nm) of 0.4 in approximately 5–6 h from initial O.D. of 0.02 in MOPS media (Supplementary Figure S2). There was a slight growth inhibition on DMS and CHP treatment during mid-log growth at concentration of 0.1%. We have regarded this inhibition as non-significant and have chosen this concentration for subsequent RNA extraction. Previous work using *E. coli* to study the genes involved in tolerance (using microarray/genomic library screening) of selected VOCs uses a range of concentrations from 0.5%^36^ to 1.7% butanol^36^, and up to 10% for toluene^29^. The concentration of n-butanol that caused a 50% growth decrease in M9 medium in *E. coli* DH1 was 0.8%(v/v)^33^. Most of these studies used concentrations that are growth inhibitory to *E. coli*. We expect that the transcriptome of *E. coli* using non-inhibitory levels of compounds used in the current study would provide new insights compared to existing literature.

**Figure 1:**
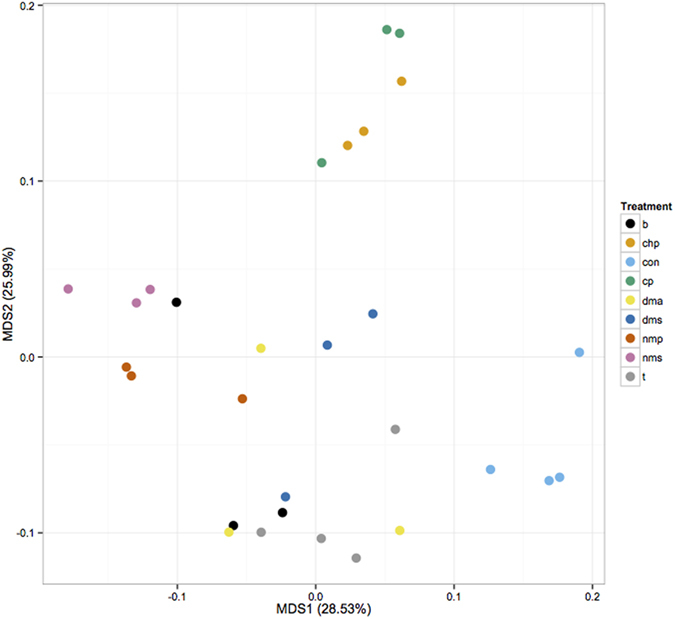
Multi-dimensional scale (MDS) plot of the global gene expression profiles of *E. coli* samples based on normalized feature count values. B: n-butanol; chp: N-cyclohexyl-pyrrolidone; con: no VOC control; cp: cyclopentanone; dma: N,N-dimethylacetamide; dms: dimethyl sulphide; nmp: 1-methyl-2-pyrrolidone; nms: N-methyl succinimide; t: toluene.

**Figure 2:**
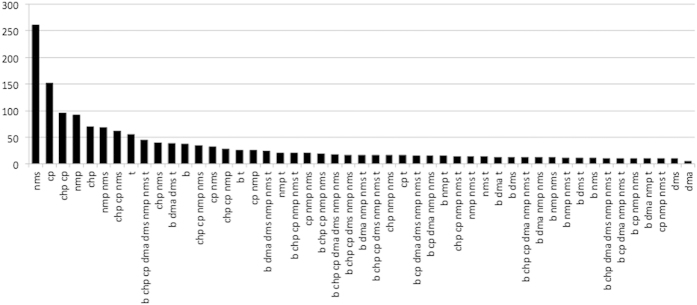
Number of differentially expressed genes based on Venn analysis of eight different treatments. Numbers of genes responsive only to single treatment are reported for all eight VOCs. For genes that were shared between two or more treatments, only those with number of shared genes exceeded ten were plotted.

**Figure 3:**
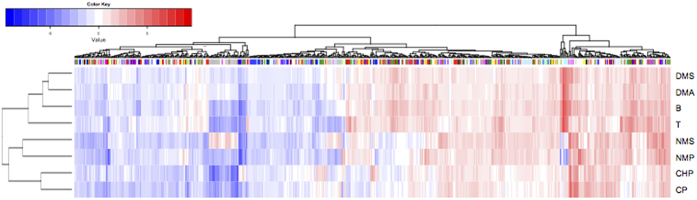
Heatmap of logFC values of 625 DE genes that occurred in at least four VOC treatments in *E. coli*’s transcriptome. Color bar on column dendrogram indicate COG functional category of the DE genes. Only COG categories containing more than 5 DE genes are colored. COG category with no known function, such as “R” and “S” are not colored. Color key: Green: Energy production and conversion [C]; Red: Amino acid transport and metabolism [E]; light green: Nucleotide transport and metabolism [F]; Blue: Carbohydrate transport and metabolism [G]; Orange: Coenzyme transport and metabolism [H]; Light purple: Lipid transport and metabolism [I]; Light blue: Translation, ribosomal structure and biogenesis [J]; Magenta: Transcription [K]; Pink: Replication, recombination and repair [L]; Maroon: Cell wall/membrane/envelope biogenesis [M]; Grey: Cell motility [N]; Black: Posttranslational modification, protein turnover, chaperones [O]; Yellow: Inorganic ion transport and metabolism [P]; Light orange: Secondary metabolites biosynthesis, transport and catabolism [Q]; Cyan: Signal transduction mechanisms [T]; Olive: Defense mechanisms [V].

In the present transcriptomic analysis, read mapping against the *E. coli* K-12 MG1655 genome was performed which allowed us to identify differentially expressed genes. The analysis identified the expression of 4140 coding DNA sequence (CDS) tags. The non-metric multidimensional scaling (NMDS) plot of global mRNA expression profiles revealed separate clustering patterns on cells grown with VOC compared to the no VOC controls, with NMS and NMP-treated cells clustering furthest from the controls on the first dimension (Fig. 1). Biological replicates for most VOC treatments clustered tightly indicating consistency between the replicates, although the clustering for treatment DMS, DMA, B are not as tight compared to the rest of the treatments. The differentially expressed (DE) genes identified (with cut off at log fold change of greater than 1 or less than −1, an average logCPM value of greater than or equal to 3, and a *p*-value less than 0.05) are distributed across a range of average logCPM values (Supplementary Figure S3). More DE genes were up rather than down regulated following treatment by B, DMA, DMS, and T. The converse was true for CHP, CP, NMP, and NMS-treated cells (Supplementary Figure S3, Table 2). The percentages of genes identified as significantly differentiated over the total gene expression profile in VOC treatments compared to the controls ranged from 9.28% (DMA) up to 25.94% (NMS) (Table 2). Similar trend was found for chemical-specific gene responses (identified based on Venn analysis of DE genes), with DMA having the lowest (1.30%) and NMS the highest percentage (24.21%) (Table 2, Fig. 2). In addition, a total of 625 DE genes were shared by four or more VOC treatments, suggesting a subset of common genetic responses. The expression pattern for these DE genes appears to be divided into two major clusters for the VOCs used in this study (Fig. 3). Cells grown with B, T, DMS and DMA elicited more similar transcription patterns than CHP, CP, NMP and NMS. (Fig. 3). These observations suggest that some VOCs might induce more cellular responses compared to others at non-growth inhibitory concentrations. Clustering of the overall transcriptome patterns of VOC treatments (Fig. 1) had some resemblance compared to the shared DE gene profiles (Fig. 3). For example, the profile of treatment CHP and CP, NMP and NMS are clustering closer to each other compared to other treatment in both the MNDS and heatmap plots. The relationship between the chemical properties of the compounds tested and the degree of cellular response in *E. coli* would be an interesting investigation in the future.

A number of genes relating to cold-shock responses were up regulated in our transcriptomic datasets (Supplementary Table S1). We have disregarded these genes as response to VOCs as the promoter clones for these genes failed to show an increase in GFP expression compared to the control in our bioassays at 37 °C (Supplementary Figure S4). These cold-shock related genes are likely to be an artifact of concentrating the biomass at 4 °C.

### Functional gene categories induced by multiple chemical treatments

#### Induction of iron-sulfur assembly system

Fe/S proteins participate in diverse biological processes such as respiration, central metabolism, DNA repair and gene regulation^40^. The iron-sulfur cluster (ISC) and sulfur mobilization (SUF) systems carry out biogenesis and maturation of all Fe/S clusters in prokaryotes. In the ISC system, IscU and IscS are required to build the Fe/S cluster, followed by release of Fe/S cluster by HscA and HscB. In the SUF system, SufSE forms the Fe/S cluster, and SufBCD complex is responsible for cluster transfer and release^40^. The compounds used in the current study had a higher expression of genes under different Fe/S cluster biogenesis system compared to the no chemical treatment control. Both ISC and SUF systems were activated following B and CP treatment, while only the ISC system is activated following CHP, NMP, NMS and T treatment, and only SUF system was activated when cells were grown with DMA and DMS (Table 3). This suggests that different chemicals induced distinctive responses in Fe/S assembly systems. *IscR*, a gene encoding the regulator responsible for Fe/S homeostasis and regulates the expression of a number of Fe/S proteins^41^, was also up regulated in cells exposed to the eight compounds tested in the current study. The up regulation of *iscR* is validated through promoter: GFP fused expression assays (Supplementary Figure S5). IscR represses its own expression when there is sufficient Fe/S cluster in the cell, and the *isc* operon is activated when cells are under Fe/S cluster-limiting and oxidative stress conditions. Overexpression of *iscR* might indicate that the chemicals tested in the current study elicited an oxidative stress or iron-limiting condition on the cells. This could be caused by the action of the VOCs on outer membrane proteins^33^. In addition, the SUF system is believed to provide better resistance to iron^40^,^42^ and oxidative stresses compared to the ISC system^43^,^44^,^45^. Whether the induction of the SUF system when cells were exposed to DMA and DMS is directly linked to oxidative stress is unknown, as other regulators, like Fur, OxyR are also known to be involved in SUF-type Fe/S regulation^40^.

#### Oxidative stress responses

A number of genes known to be induced by oxidative agents were up regulated in response to at least 4 VOCs used in the current study (Table 3 and Supplementary Figure S5). PqiAB is a SoxRS-regulated membrane protein known to be induced by paraquat and other superoxide generators, but it is not induce by hydrogen peroxide, ethanol and heat shock^46^. YhcN was identified as a stress protein associated with hydrogen peroxide, cadmium and acid^47^. MntS confers resistance to hydrogen peroxide by facilitating delivery of Mn^2+^ to Mn^2+^-dependent enzymes^48^. A gene encoding for methionine sulfoxide reductase, *msrB*, was up regulated as well. MsrB repairs methionine residues in proteins that have been oxidized by reactive oxygen species^49^. Collectively, the results indicate that *E. coli* cells exposed to the compounds tested in the study induce oxidative stress responses even at non-inhibitory concentrations. In addition, there might be proteins oxidized by the presence of VOCs. *yfbU*, a gene known to be involved in cell death by oxidative DNA damage^50^, was down regulated in all treatments, suggesting that the cells did not go through the toxin:antitoxin response when grown with chemical tested, but instead employ alternative oxidative stress responses as described.

#### Induction of various transporter proteins

**Figure 4:**
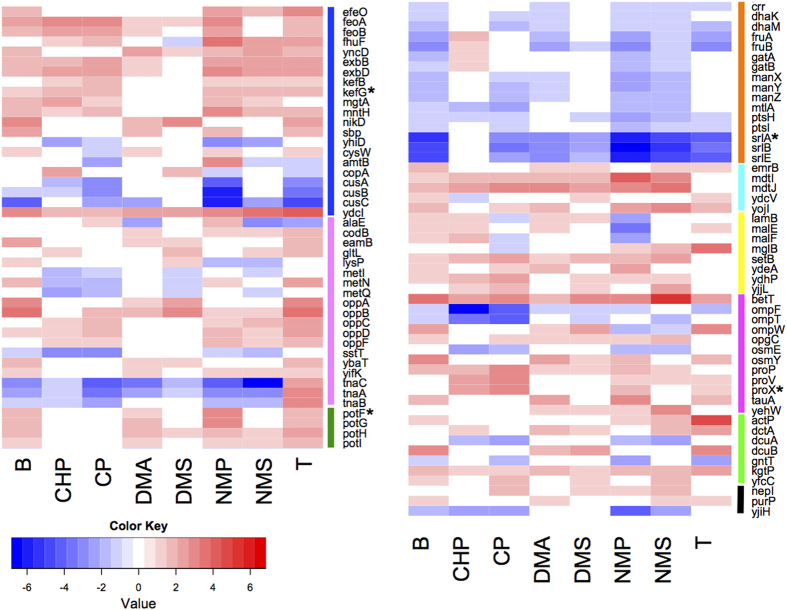
Heatmap of log fold-change values of transporter genes. Type of transporter proteins identified as differentially expressed in at least 4 VOC treatments. Color code for transporter type: blue: inorganic ion; pink: amino acid; green: putrescine; orange: PTS; cyan: multi-drug efflux; yellow: sugar; magenta: osmosis; lime: organic acids; black: others. Cells in white are values considered as insignificant (*p*-value <0.05). Genes marked with “*”: Gene promoter-fused GFP assays performed (Supplementary Figure S5).

Transporter proteins for inorganic ions, amino acids, and the PTS systems were among the top three categories to be differentially expressed in at least 4 chemical treatments compared to the control (Fig. 4 and Supplementary Figure S5). Genes involved in the uptake of both inorganic iron (e.g. *feoA, feoB* and *efeO*), and siderophores (*exbBD*, *yncD* and *fhuF*) were up regulated. Genes involved in iron uptake have been shown to increase *E. coli*’s tolerance to environmental stresses. For example, over expression of *feoA* increases the tolerance of *E. coli* to butanol^36^, and *efeO* confers resistance to mitomycin C and other stresses such as UV irradiation compared to wild type cells^51^. ExbB and ExbD proteins are required to provide energy for the import of iron-siderophore complexes and vitamin B12 across the outer membrane via TonB^52^,^53^,^54^. YncD, a putative TonB-dependent outer membrane transporter for iron^55^, could be one of the protein targets of TonB-ExbB-ExbD. The FhuF protein is required for cells to use hydroxamate-type siderophores as iron source^56^. Collectively, up regulation of iron uptake genes implies that the cells are actively utilizing iron, possibly for the formation of Fe/S cluster containing proteins as described above.

Transporters for other inorganic ions were also up regulated (Fig. 4), e.g., genes for magnesium (*mgtA*) and manganese (*MntH*) uptake. MntH was to shown support the growth of *E. coli* cells encountering iron-deficiency and oxidative stress^57^. During H_2_O_2_ stress, mutants lacking ability to import manganese and iron suffer high rates of protein oxidation, implying the role of MntH in preventing protein damage. Potassium efflux genes (*kefB* and *kefG*) were up regulated too. Efflux of potassium is known to play a role in protecting the cell from electrophile toxicity through acidification of the cytoplasm^58^, suggesting cells grown with VOC might be undergoing electrophilic stress.

The second largest transporter type relates to amino acids (Fig. 4). In particular, the dipeptide ABC transporter, encoded by the *oppABCDF* operon, was up regulated in most VOC treatments. The OppABCDF system function in oligopeptide uptake as well as recycling of cell wall peptides^59^. Expression of *opp* genes was up regulated in cells treated with 1% isobutanol as an early stage response^38^, and *oppD* increased antibiotic resistance in *E. coli* during biofilm formation^60^. Increased expression of the *opp* genes support previous findings that these transporters are involved in VOC resistance. The *tnaCAB* gene cluster, responsible for the uptake of tryptophan, was down regulated in response to most VOC used. Mutants lacking *tnaCAB* had increased isobutanol tolerance^61^, supporting our finding that tnaCAB plays a negative role in VOC tolerance. The cytoplasmic putrescine transporter protein, encoded by *PpotFGHI*, was significantly up regulated following n-butanol, DMA, NMP and T treatment. The up regulation of *potG* stimulates cell growth in the presence of phenylpropanoids, which indicates that PotFGHI might also be involved in the import of this compound class^62^. Cells grown with VOCs could either have an elevated concentration of putrescine inside the cell, or could also plays a role in transport of VOCs.

The third most abundant transporter class containing DE genes identified belong to the phosphotransferase (PTS) system, which is an active transport system responsible for uptake of nutrients in bacteria (Fig. 4). The PTS system is activated when ambient nutrient level is low^63^. In this study, most of the DE genes under the PTS systems were down regulated in most VOC treatments, including those responsible for glucose, dihydroxyacetone, fructose, galactitol, mannose and glucitol. Down regulation of these systems could be explained by the high nutrient media utilized in growing the cells (1.5% glucose), hence the cells does not require active transport for nutrient uptake.

Other transporter types with differential gene responses include multidrug efflux proteins and those related to osmotic response (Fig. 4). Three genes related to multidrug efflux proteins, *mdtI*, *mdtJ* and *emrB*, were up regulated in most chemical treatment used in the current study. MdtJ and I are two components of a spermidine exporter^64^ and emrB is known to increase tolerance to hydrophobic compounds, such as organomercurials and nalidixic acid^65^ and thiolactomycin^66^. Multidrug exporters are capable of exporting compounds consisting different structural components, hence they could potentially export the compounds tested in the current study. Genes known to be associated with maintaining appropriate osmotic conditions in cells, for example, *osmY*, and ABC transporters for transport of osmoprotectants like proline, glycine betaine, and taurine (*proP*, *proV*, *proX* and *tauA*) were up regulated. The VOC used in the current study might have an effect in the osmotic condition in *E. coli* cells, hence inducing the expression of this gene class. In addition, the expression of a DNA-binding transcriptional repressor known to confer organic and inorganic acid stress, *ydcI*, was up regulated in all VOC treatment. YdcI protein is conserved across gram-negative bacteria and a *S. typhimurium* mutant lacking this gene had decreased resistance to acid stress^67^. Up regulation of *ydcI* genes in our study imply that this gene may also be a response to VOC.

#### Universal stress proteins

*E. coli* harbors six *usp* genes – *uspA*, *C*, *D*, *E*, *F* and *G*.^68^,^69^. The functions of Usps overlap to some extent, e.g. both UspA and UspE are involved in oxidative stress defense^68^, while UspG and UspF are associated with fimbriae-associated adhesion^68^,^70^. From the transcriptomic results of the current study, we observed a down regulation of *uspA* and *uspG* in most VOC treatments, while *uspE and uspF* were up-regulated in B, DMA, DMS and T (Table 3, Supplementary Figure S5). As UspA have functions that overlaps with UspE, down regulation of *uspA* can be compensated for by the expression in *uspE*. Similarly, down regulation of *uspG* expression can be compensated by up regulation of *uspF*.

#### Flagella and cellular motility

Many genes relating to flagella biosynthesis and motility (the *flg*, *flh* and *fli* genes) were significantly down regulated in all VOC treatments, with the exception of treatment NMS (Table S2). Previous studies have found that flagellar biosynthesis was down regulated in *E. coli* exposed to ethanol^30^ as well as heat stress^71^. Since NMS is not a VOC, it is not surprising that these genes were not repressed. However, a decrease in expression of flagella genes did not result in a reduction in motility in soft agar motility assays (Supplementary Methods and Figure S6). It is possible that the *E. coli* cells have already synthesized the flagellum before flagellar gene repression occurring in the assay. Other possible reasons include the differences in growth condition of *E. coli* due to the nature of the motility assay, e.g. surface-associated soft agar versus liquid, and the time of incubation.

### Functional gene categories induced by specific chemical treatments

#### Shared DE genes responsive to CHP and CP

**Figure 5:**
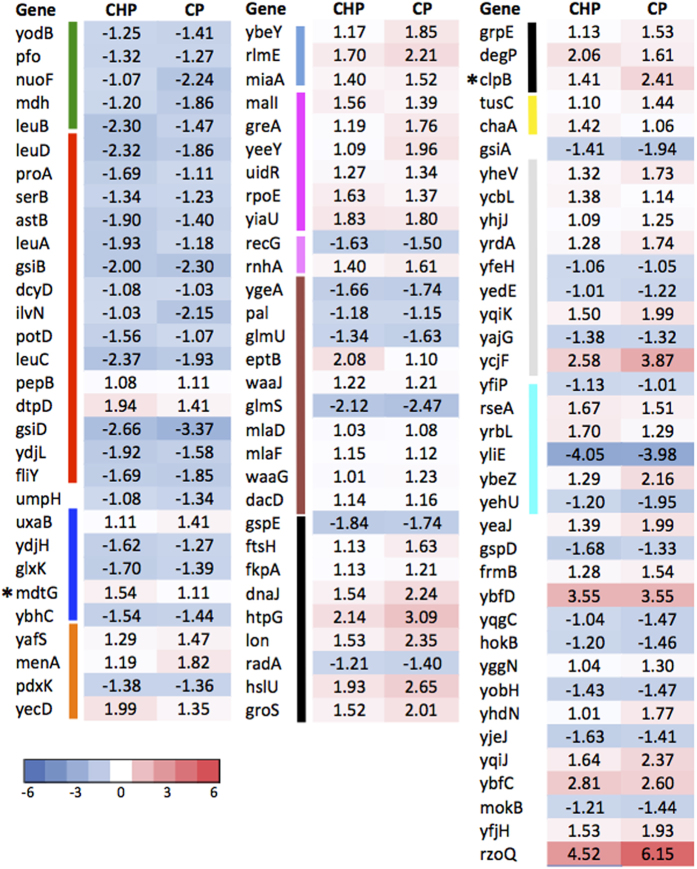
Log fold change values of the 96 shared DE genes between cells exposed to CHP and CP treatment. Color bars beside gene names indicate COG categories (labeling same as Fig. 4, except that COG category “R” and “S” are labeled as light grey). Gene name with “*”: expression tested on promoter:GFP fused *E. coli* clones.

A total of 96 genes responded significantly with specificity to CP and CHP, which shared the highest number of genes compared to other chemical treatment combinations (Figs 2 and 5). Top COG categories of the shared DE genes belong to Post-translational modification, protein turnover and chaperones (O), Amino acid transport and metabolism (E), Cell wall/membrane biogenesis (M) (Fig. 5). A few DE genes identified gave indications that CP and CHP might interfere with protein structure and outer membrane integrity. For example, the *mlaD* and *mlaF* genes, which prevent accumulation of phospholipids (PLs) in the outer leaflet of the outer membrane in *E. coli* cells, were up regulated. Cells accumulate PLs in the outer leaflet of the OM when exposed to harsh chemical treatments. This process would disrupt the LPS organization and increasing sensitivity to small toxic molecules^72^. Up regulation of *mla* genes imply that the cells’ OM lipid asymmetry could be disrupted in the presence of the chemicals tested. In addition, a number of genes encoding for molecular chaperones were significantly up regulated in response to CHP and CP. These include the protein (re)-folding chaperones (*htpG, fkpA*, *dnaK-DnaJ-GrpE* and the *GroES*), protein resolubilization chaperones (*clpB*) and a protease involving in clearing the defective peptides (*hslU*). Up regulation of these genes imply that CHP and CP cause cellular protein misfolding in *E. coli*.

Transporter-related genes specifically up regulated in response to CHP and CP include genes encoding for peptide transport proteins (*dtpD*), and a putative drug efflux system protein (*mdtG*). Overexpression of *mdtG* has been found to increase resistance to deoxycholate (bile acid) and the board spectrum antibiotic fosfomycin^73^. Up regulation of such multidrug efflux genes could imply that cells perceive CP and CHP compounds as drugs and attempt to export them out of the cells.

#### Shared DE genes responsive to NMP and NMS

**Figure 6:**
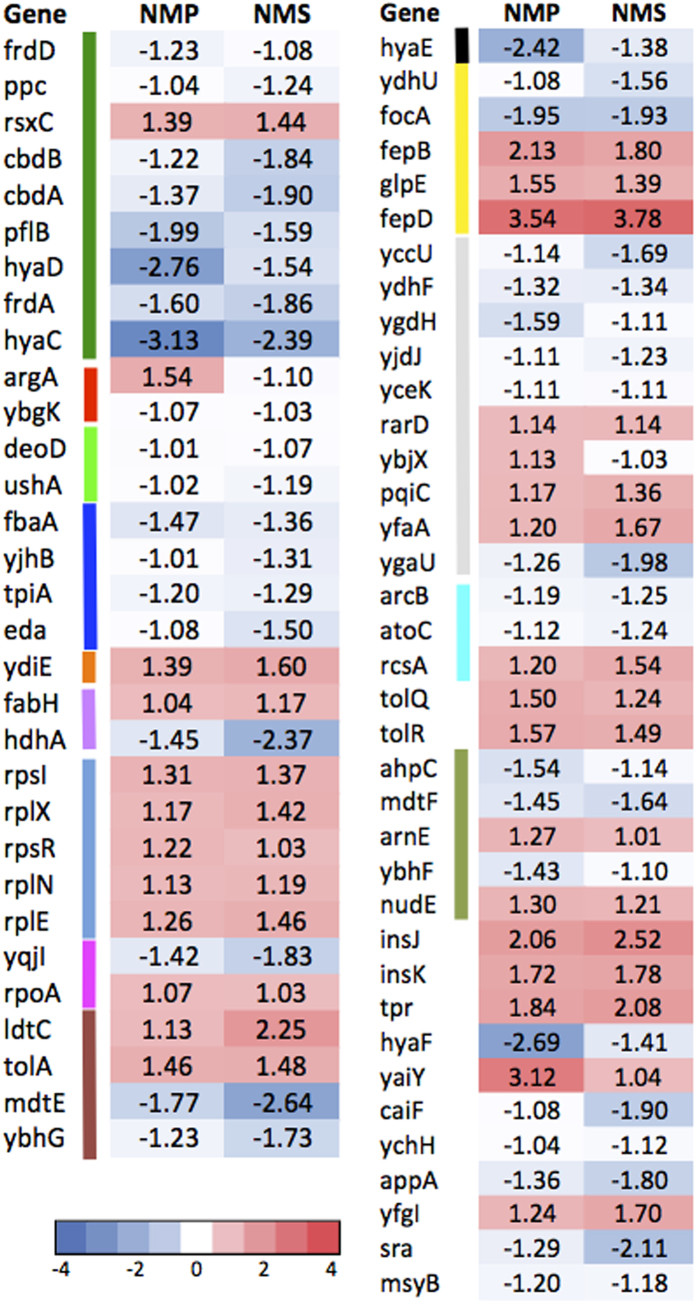
Log fold change values of the 68 shared DE genes between cells exposed to NMP and NMS treatment. Color bars beside gene names indicate COG categories (labeling same as Fig. 4, except that COG category “R” and “S” are labeled as light grey).

The next chemical pair sharing the highest number of DE genes is NMP and NMS, sharing 68 genes based on Venn analysis (Figs 2 and 6). NMP is an organic compound consisting of a 5-membered lactam and NMS is a metabolite of NMP biodegradation^9^. Although NMS is not considered as a VOC, it is cyclic. Most DE genes under energy production and conversion responding specifically to NMP and NMS were down regulated (e.g. *hyaDC*, *cbdAB* and *frdAD* genes), except for *rsxC*, which is part of the *rsx* operon (Fig. 6). The *rsxABCDGE* gene cluster is involved in switching off the SoxR-mediated induction of SoxS transcription factor when cells are deficient of oxidizing agents^74^. Up regulation of these genes could imply that the cells cultured with NMP and NMS were less prone to oxidative stress and require SoxR reduction to repress downstream activation of SoxS. Interestingly, *rxsA* was shown to be important for survival of cells exposed to ionizing radiation^75^.

Genes encoding for TolA-TolQ-TolR complex, were up regulated in cells treated with NMP and NMS (Fig. 6). The Tol-Pal cell envelope complex is known to be involved in maintaining cell envelope integrity, and mutants have greatly increase sensitivity to drugs and detergents and are prone to periplasmic leakage^76^,^77^. Cells treated with NMP and NMS might respond differently to membrane disruption compared to that of CP and CHP. NMP and NMS activate the TolAQR complex whereas cells exposed to CP and CHP activate the Mla pathway. The molecular mechanisms behind activation of different gene clusters in response to maintenance of cell envelope integrity would be an interesting area for future investigations.

Genes under “Defense mechanisms” that were up regulated include *arnE* and *nudE* which belong to the drug/metabolite transporter superfamily and the Nudix hydrolases family respectively (Fig. 6). Genes relating to iron-enterobactin transporter (*fepB* and *fepD*) and thiosulfate: cyanide (*glpE*) sulfurtransferase were up regulated specifically following NMP and NMS treatment. The *fepBCDG* complex together with the TonB-dependent outer-membrane transporter, and *fepA*, is responsible for the import of ferric enterobactin across the cell envelope. In addition to the iron-uptake system discussed in the previous sections, cells treated with NMP and NMS appear to have an additional iron-enterobactin transporter up regulated in the conditions tested in this study.

#### Stress and membrane repair-related DE genes responsive to one chemical treatment

**Figure 7:**
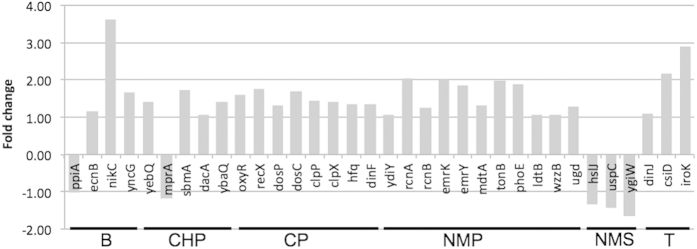
Fold change of the DE genes related to stress response and membrane repair induced by specific VOCs.

Genes that responded positively to one particular VOCs were identified, with a number of them related to stress (*oxyR*, *dinF*, *ydiY*), transport pumps for metals (*nikC, rcnA* and *rcnB*) and transport pumps for drugs (*emrKY*, *mdtA*, *sbmA*, *yebQ*) (Fig. 7). Expression of *emrK* (part of the EmrKY-TolC multidrug efflux transport system) was found to increase in the presence of sub-inhibitory concentration of a number of antibiotics^78^. As the concentration of the chemical used in this study is considered non-inhibitory, results supported the conclusion that low concentrations of compounds are sufficient to induce a transcriptional response in various functional categories from the cell (Supplementary Figures S7). A number of genes relating to cell wall biogenesis were specifically up regulated when cells were exposed to NMP (*tonB*, *phoE*, *ldtB*, *wzzB*, *ugd*). Induction of these genes could imply that the cells have membrane component biosynthetic pathways activated specifically when exposed to NMP, implying that the involvement of NMP damages cell wall component, thus requiring repair.

#### COG category enrichment of DE genes

**Figure 8:**
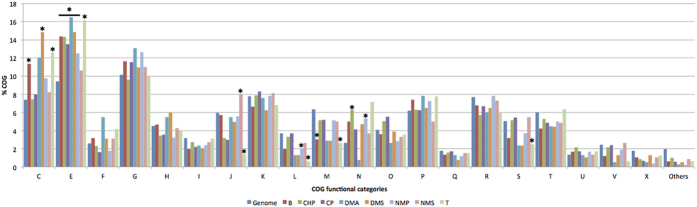
Percentage distribution of COG categories of the DE genes under selected VOC treatments. Asterisks marked the COG categories in treated samples that had a significantly different COG distribution from the background composition of the *E. coli* genome (with confidence level of 0.99 and bootstrap replicates of 10000). Key for the various COG functional categories are as described in Fig. 4, with additional categories as follows: U: Intracellular trafficking, secretion, and vesicular transport; X: mobilome, phages, and transposons.

We performed COG enrichment analysis of total DE genes induced by individual chemical treatments against the *E. coli*’s genome copy of COG distribution (Fig. 8). More than half of the chemicals tested had amino acid related genes overrepresented compared to the *E. coli* genomic background. Amino acid metabolism is central to cellular survival and it is related to many parts of cellular metabolism. Genes under this category have been found to be differentially expressed in *E. coli* cells exposed to butanol^33^,^36^. Cells exposed to n-butanol, DMS and toluene have a significant higher number of DE genes belonging to COG category of energy conversion, implying that genes under energy conversion are responsive to these VOCs. NMS is the only treatment that had genes relating to translation overrepresented. A total of 31 genes under translation and ribosomal biogenesis category were specifically responsive to NMS, many of them encode for ribosomal subunit proteins, implying that the cells were actively synthesizing proteins. Being a metabolic by-product of NMP, NMS is not a VOC, and appears that this metabolite does not impair cellular metabolism/growth at all. Both CHP and NMP had motility gene class overrepresented compared to *E. coli*’s background as well. COG categories were under represented compared to *E. coli*’s genomic background including those related to replication and repair for treatment NMP and T, and cell wall biogenesis were underrepresented in treatment n-butanol and toluene. Collectively these results could imply that different VOCs induced genes under specific COG categories.

#### Catabolic pathways of VOCs and aromatic compounds

Little is known about the biodegradation of VOCs focused on in this study. The genome of *E. coli* K-12 contains neither the genes responsible for the degradation of DMS (e.g. DMS monooxygenase, DMS dehydrogenase and DMS methyltransferase)^79^, nor for toluene (i.e. toluene-2,3-dioxygenase)^80^. Transcriptomic profiles of genes encoding for ring-hydroxylating oxygenases and transformation of aromatic compounds revealed very few differentially expressed genes in cells treated with the VOCs in the current study, with the exception of *tnaA* and *entA*, which were up-regulated following toluene treatment, and *ubiX*, *ubiB*, which were up-regulated with CHP and NMS treatment (Supplementary Table S3). However, these genes are also involved in generic cellular metabolism and their direct involvement in the transformation of VOCs in this study is yet to be determined. A search for xenobiotics degradation pathways in KEGG (according to which some pyrrolidones have been classified), revealed that most of the described xenobiotics in KEGG are structurally very different from the VOC used here. Hence to the best of our knowledge, this study is the first to describe transcriptomic responses of *E. coli* K-12 exposed to VOCs with pyrrolidone backbone.

In conclusion, RNA-seq data in this study suggested that a variety of genes relating to Fe/S cluster biogenesis, oxidative and universal stress responses, as well as transport and membrane bound proteins are responsive to selected VOCs in *E. coli.* These genes were differentially expressed when the cells were in balanced-growth and at the highest non-inhibitory concentrations, which is well above the basal detectable environmental levels (PUB, personal communications). By identifying the transcriptional responses occurring between the basal levels and high concentration spikes, we have set the framework for the analysis of the dose dependent response, a key element in biosensor development. The numerous changes in gene expression upon exposure to the different VOCs suggests that *E. coli* might exhibit analogous response when exposed to chemical compounds of similar nature. It is interesting to speculate that the clustering of DE genes in response to different VOC tested could be related to the overall physical properties (polarity, volatility) and to the structure of the VOCs (i.e. linear chain vs cyclic compounds) used in the current study. Further studies are necessary to uncover the specific molecular mechanisms of *E. coli*’s cellular responses to chemical compounds of different structures. In addition, a number of DE genes described in this study, for example, those related to Fe/S cluster biogenesis, and various transporter genes, are conserved in other environmentally relevant bacteria, such as *Pseudomonas* species^20^. Results from the current study hence could also be applicable to future biosensor development in bacteria other than *E. coli*. However, one should note that some *Pseudomonas* species are known to be able to metabolize a number of VOCs and cyclic hydrocarbons via enzymatic conversions^22^,^81^, hence their global genetic response to VOCs might be different from *E. coli*.

## Experimental Procedures

### Chemicals

Chemicals used were as follows: n-butanol (B), N-cyclohexyl-pyrrolidone (CHP), Cyclopentanone (CP), N,N– Dimethylacetamide (DMA), Dimethyl sulphide (DMS), N-methyl-2-pyrrolidone (NMP), and N-methyl-succinimide (NMS) and Toluene (T). All were purchased from Sigma-Aldrich (Taufkirchen, Germany) and were of analytical purity.

### *E. coli* cultivation and RNA extraction

*E. coli* K-12 strain MG1655 was cultured in 10 mL LB5 broth within a shaking incubator at 150 rpm and at 37 °C for 16 h. The overnight culture was diluted (1:100) in 10 mL MOPS medium (Neidhardt *et al.* 1974) supplemented with 1.5% glucose. Based on the MIC assays (Supplementary Methods), different VOC concentrations were added at the beginning of cultivation (Table 1) and three biological replicates were used for each chemical treatment. Cells were grown in Balch-type tubes (18 × 150 mm) with 20 mm butyl rubber stopper and aluminum seal to minimize leakage of VOCs during the cultivation time. Cells were incubated in a shaking incubator at 37 °C and were harvested for RNA extraction when OD_600_ reached 0.4. The RNA extraction was as follows: 5 mL aliquots of the cultures were added to two volumes of RNAprotect Bacteria Reagent (Qiagen). The mixture was incubated at room temperature for 5 min followed by centrifugation at 4,000 × g for 10 min at 4 °C. The supernatant was removed and the cell pellets were stored at −80 °C until RNA extraction. RNA was extracted using the RNeasy® Mini Kit (Qiagen), following the manufacturer’s recommendations. Contaminating DNA was removed using DNsae (Qiagen) until DNA concentration was less than 5% of the RNA. DNA and RNA concentrations were measured using Picogreen and Ribogreen assays (Invitrogen), respectively.

### RNA sequencing

The quality of the RNA samples was determined by running the samples on a Bioanalyzer RNA 6000 Pico Chip (Agilent). Next-generation sequencing library preparation was performed following Illumina’s TruSeq Stranded mRNA Sample Preparation protocol with the following modifications: RNA samples were added to the elute-fragment-prime step. The PCR amplification step, which selectively enriches for library fragments that have adapters ligated on both ends, was performed according to the manufacturer’s recommendation. Each library was uniquely tagged with one of Illumina’s TruSeq LT RNA barcodes to allow library pooling for sequencing. Library quantitation was performed using Invitrogen’s Picogreen assay and the average library size was determined by running the libraries on a Bioanalyzer DNA 1000 chip (Agilent). Library concentrations were normalized to 2 nM and validated by qPCR on a ViiA-7 real-time thermocycler (Applied Biosystems), using qPCR primers recommended in Illumina’s qPCR protocol, and Illumina’s PhiX control library as standard. Libraries were then pooled and sequenced in one lane of an Illumina HiSeq2500 rapid sequencing run at a read-length of 101bp paired-end. Sequencing data have been submitted to GenBank SRA archive with the BioProject ID: PRJNA286974 and SRP accession SRP059483.

### RNAseq data analysis

Quality trimming and adaptor removal were done using Cutadapt v1.9.0^82^ with the following parameters: –q 20, –m 30, –overlap 10, –quality-base 33. Sequences were mapped to the *E. coli* str. K-12 MG1655 genome (NCBI accession: NC_00913.3) by bowtie2^83^ with end-to-end and very-sensitive modes. The alignments were converted to .bam and .bam-indexed files using Samtools^84^. Sorted alignment files were imported into R to calculate overlapping reads as counts per gene using a combination of the following R packages: Rsamtools, GenomicFeatures and GenomicAlignments^85^. Only the concordant pairs in the sorted *.bam files were imported using the function “readGAlignmentPairsFromBam”. Differential genes were identified from the tabular output of gene count abundance using edgeR package^86^. The edgeR package implements a quantile-adjusted conditional maximum likelihood (qCML) estimator for the dispersion parameter of the negative binomial distribution^86^,^87^. Testing for DE genes from biological replicates is based on the exact test derived based on these models. To calculate differentially expressed genes, all VOC treatments were compared to the control in which the cells did not have any exposure to VOCs. DE genes that have at least 2-fold change, *p*-value less than 0.05 and logCPM value greater than 3 were considered significantly different from the no VOC control. Genes were mapped to COG and KEGG IDs using the December 2014 release of COG database^88^ and June 2013 release of the KEGG database (Kanehisa Laboratories). Principal component analysis (PCoA), Venn and heatmap analysis were performed using R packages (vegan, venn, heatmap.2, respectively), and pathway maps were plotted using iPATH^89^.

### GFP kinetics using fluorescent transcriptional reporter *E. coli* clones

Selected *E. coli* clones with transcriptional fusions of GFP to relevant promoters of the identified DE genes were used to validate the RNAseq results^27^. Reporter strains were inoculated from frozen stocks into 2× LB broth and incubated for 16 h at 37 °C. The cells were diluted (1:100) into fresh 1× MOPS medium supplemented with 25 μg/mL kanamycin and 1.5% glucose and grown as described previously. The VOC were added at the same concentration used in RNA experiments. When the OD_600nm_ reaches 0.35, an aliquot of culture was transferred to 96-well microplate. Optical densities were measured at OD_595nm_ and GFP intensity were measured at 485/535nm at 15 min interval for 4 h. Triplicates were performed and cells grown without VOC were used for comparison. *E. coli* clone with the same vector backbone without any promoter was used as background noise subtraction. Fold-change analysis was performed and maximum fold-change was recorded.

## Additional Information

**Accession codes:** Sequencing data have been submitted to GenBank SRA archive with the BioProject ID: PRJNA286974 and SRP accession SRP059483.

**How to cite this article**: Yung, P. Y. *et al.* Global transcriptomic responses of *Escherichia coli* K-12 to volatile organic compounds. *Sci. Rep.* **6**, 19899; doi: 10.1038/srep19899 (2016).

## Supplementary Material

Supplementary Information

## Figures and Tables

**Table 1:** Basic properties of the VOCs used in the study.

Chemical	Linear formula	MW	v/v%	ppm	mM	logP_ow_	V (Pa)	Melting	Boiling
n-butanol (B)	CH_3_(CH_2_)_3_OH	74.12	0.1	1000	13.49	0.88	1200	−89.9	117.7
N-cyclohexyl-pyrrolidone (CHP)	C_10_H_17_NO	167.25	0.1	1000	5.98	1.41	6.7	12	284
Cyclopentanone (CP)	C_5_H_8_(=O)	84.12	0.1	1000	11.89	0.7	1520	−58	131
N,N-dimethylacetamide (DMA)	CH_3_CON(CH_3_)_2_	87.12	0.5	5000	57.39	−0.7	300	−20	165
Dimethyl sulphide (DMS)	(CH_3_)_2_S	62.13	0.1	1000	16.1	0.92	53700	−98	35–41
1−methyl-2-Pyrrolidone (NMP)	C_5_H_9_NO	99.13	0.1	1000	10.09	−0.46	42	23–24	201
N-methyl succinimide (NMS)	C_5_H_7_NO_2_	113.11	0.5	5000	44.2	NA	NA	65	235
Toluene (T)	C6H5CH3	92.14	0.02	200	2.17	2.69	2800	−95	110.6

MW: Molecular weight; v/v% concentration in volume to volume ratio used for RNAseq based on a semi-MIC quantification assay; ppm: parts per million; mM: milliMolar; logP_ow_ value: the logarithm of partitioning coefficient in a defined octanol-water mixture values; V (Pa): Vapor pressure in Pa; Melting: melting temperature (°C); Boiling: boiling temperature (°C); NA: Not available.

**Table 2:** Summary of total the number of genes significantly up- or down-regulated (Up-/Down- all), and those responsive to specific VOCs in *E. coli*’s transcriptome (Up-/Down- specific).

Sample	Up-all	Down-all	Sum-all	Up-specific	Down-specific	Sum-specific	% Up-all	% Down-all	% Sum-all	% Up-specific	%Down specific	% Sum specific
B	430	249	679	25	12	37	10.39	6.01	16.40	3.68	1.77	5.45
CHP	324	388	712	33	37	70	7.83	9.37	17.20	4.63	5.20	9.83
CP	403	494	897	87	64	151	9.73	11.93	21.67	9.70	7.13	16.83
DMA	261	123	384	4	1	5	6.30	2.97	9.28	1.04	0.26	1.30
DMS	274	120	394	7	3	10	6.62	2.90	9.52	1.78	0.76	2.54
NMP	445	406	851	71	21	92	10.75	9.81	20.56	8.34	2.47	10.81
NMS	506	568	1074	113	147	260	12.22	13.72	25.94	10.52	13.69	24.21
T	418	223	641	39	17	56	10.10	5.39	15.48	6.08	2.65	8.74

Sum: Sum of Up- and Down- regulated genes per treatment. “%**-**all” indicates the percentage of DE genes out of the 4140 locus tag analyzed, “%-specific” indicates the percentage of DE genes specific to the VOC out of the sum of DE genes of the particular VOC treatment [e.g. % Up-specific = “Up-specific”/“Sum-all” x 100].

**Table 3:** Genes responsible for Fe/S cluster biogenesis, oxidative and universal stress responses.

Treatment	ID	Gene	B	CHP	CP	DMA	DMS	NMP	NMS	T	Gene description	ISC/SUF
b dms	b1679	sufE	1.49	0.71	0.80	0.79	1.02	−0.02	−0.91	0.91	Sulfur acceptor protein	SUF
b cp dma dms	b1680	sufS	1.83	0.68	1.21	1.21	1.35	0.28	0.36	0.91	Cysteine desulfurase, SufE induced	SUF
b cp dma dms	b1681	sufD	1.72	0.88	1.46	1.11	1.14	0.33	−0.16	0.71	Component of SufBCD Fe-S cluster assembly scaffold	SUF
b cp dms	b1682	sufC	1.77	0.99	1.97	1.11	1.30	0.42	−0.67	0.92	SufBCD Fe-S cluster assembly scaffold protein	SUF
b chp cp dma dms nmp t	b1683	sufB	2.71	1.54	3.12	1.87	2.02	1.44	0.11	1.77	Component of SufBCD Fe-S cluster assembly scaffold	SUF
b cp dma dms t	b1684	sufA	3.01	1.57	4.06	2.12	2.30	1.73	0.76	2.36	Fe-S cluster assembly protein	SUF
b chp cp nms	b2525	fdx	1.03	1.31	1.27	0.59	0.45	0.67	1.69	0.48	[2Fe-2S] ferredoxin	ISC
b chp cp nms	b2526	hscA	1.11	1.03	1.22	0.74	0.62	0.88	1.9	0.76	DnaK-like molecular chaperone specific for IscU	ISC
b chp cp nmp nms t	b2527	hscB	1.43	1.38	1.69	0.89	0.93	1.27	2.44	1.16	IscU-specific HscA co-chaperone Hsc56	ISC
chp cp nmp nms	b2528	iscA	0.97	1.05	1.33	0.41	0.51	1.39	1.52	0.72	FeS cluster assembly protein	ISC
b chp cp nmp nms t	b2529	iscU	1.31	1.50	1.78	0.59	0.62	1.79	2.07	1.14	Iron-sulfur cluster assembly scaffold protein	ISC
b chp cp dms nmp nms t	b2530	iscS	1.62	1.58	1.94	0.97	1.05	2.69	2.48	1.64	Cysteine desulfurase (tRNA sulfurtransferase)	ISC
b chp cp dma dms nmp nms t	b2531	iscR*	1.91	1.62	2.17	1.38	1.32	3.81	3.07	2.00	Isc operon repressor; suf operon activator	Regulator
chp cp nmp nms t	b3414	nfuA	0.66	1.31	1.71	0.5	0.23	1.75	1.41	1.32	Fe/S biogenesis protein; putative scaffold/chaperone	Fe/S carrier
b chp cp dms nmp nms	b4705	mntS	1.14	2.06	2.34	0.89	1.12	2.64	2.97	0.94	Mn(2)-response protein, MntR-repressed	Oxidative stress
b chp cp dma dms nmp nms t	b1778	msrB*	1.63	1.33	1.07	1.39	1.05	1.76	1.93	2.52	Methionine sulfoxide reductase B (EC:1.8.4.12)	Oxidative stress
b chp cp dma dms nmp nms	b0950	pqiA*	1.33	1.07	1.1	1.03	1.08	1.4	1.99	0.83	Paraquat-inducible, SoxRS-regulated inner membrane protein	Oxidative stress
b cp nmp nms	b0951	pqiB	1.14	0.8	1.05	0.75	0.71	1.19	1.5	0.77	Paraquat-inducible, SoxRS-regulated MCE domain protein	Oxidative stress
b chp cp dma dms nmp nms t	b2294	yfbU*	−1.84	−1.36	−1.18	−1.11	−1.39	−1.70	−1.99	−1.43	UPF0304 family protein; K09161 hypothetical protein	Oxidative stress
b dms nmp t	b3238	yhcN	1.92	1.11	1.39	1.39	1.6	2.25	1.26	1.69	Cadmium and peroxide resistance protein	Oxidative stress
b chp cp dma dms nmp nms t	b3495	uspA*	−1.79	−1.63	−1.49	−1.29	−1.63	−2	−3.16	−1.4	Universal stress global response regulator A	Usps
nms	b1895	uspC	0.78	0.34	0.75	−0.03	−0.24	0.61	−1.44	−0.55	Universal stress induced protein C	Usps
chp nms	b3923	uspD	−0.74	−1.23	−0.83	−0.73	−0.83	−0.58	−2.03	−0.42	Universal stress-induced protein D	Usps
b dma dms t	b1333	uspE*	7.16	1.96	1.39	5.79	6.71	−5.12	−1.81	6.96	Universal stress-induced protein E	Usps
b dma dms t	b1376	uspF	3.79	−0.09	−0.36	2.23	3.75	−6.06	−2.75	4.16	Universal stress-induced protein F, ATP-binding protein	Usps
b dma nmp nms t	b0607	uspG*	−1.48	−0.25	−0.03	−1.62	−0.88	−1.18	−2.4	−1.48	Universal stress protein UP12	Usps

*E. coli* contains the ISC and SUF Fe/S assembly system. Treatment: Chemical treatment associated with the DE genes; ID: Gene ID; the shaded cells and bolded numbers are not shown in the table. Genes marked with “*”: Gene promoter-fused GFP assays performed (Supplementary Figure S5).
