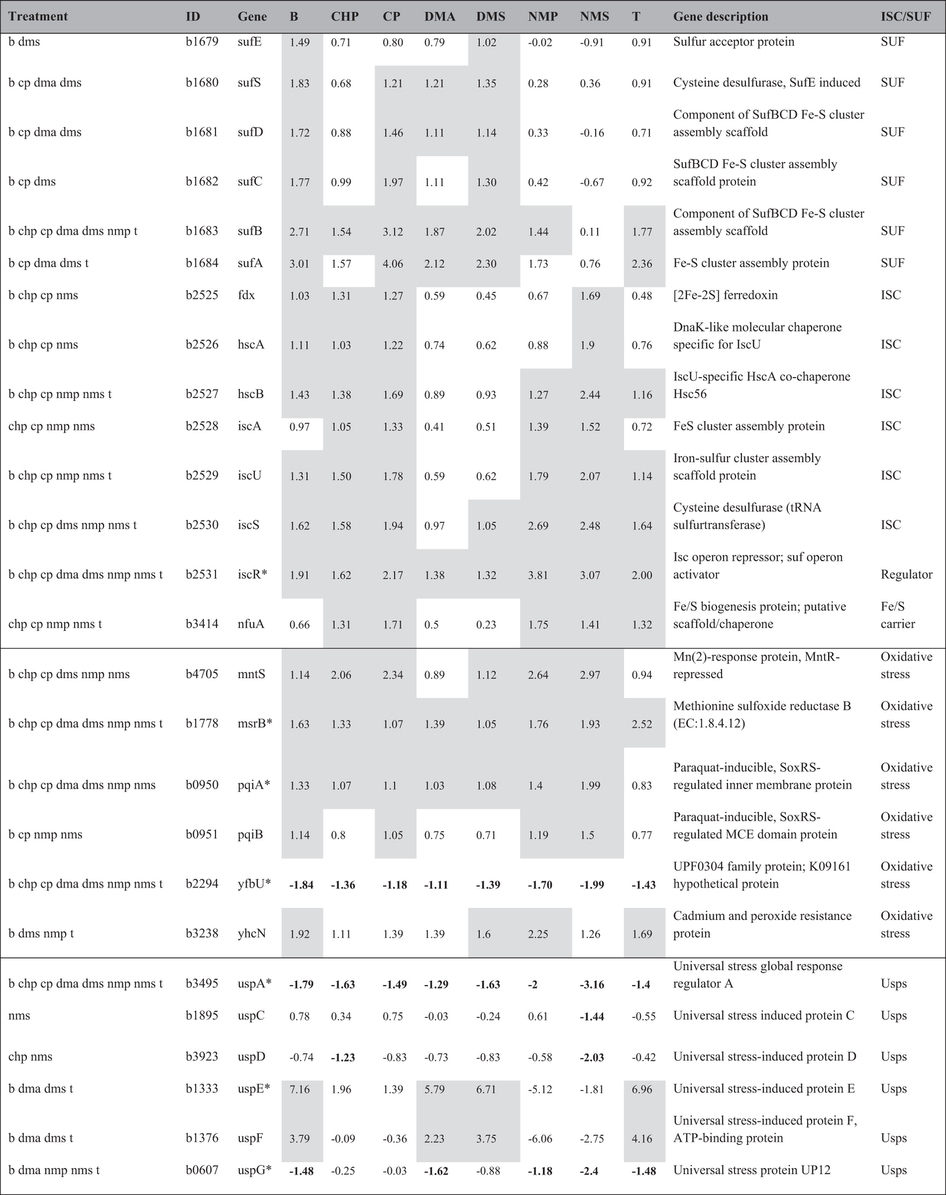# Erratum: Global transcriptomic responses of *Escherichia coli* K-12 to volatile organic compounds

**DOI:** 10.1038/srep33108

**Published:** 2017-04-06

**Authors:** Pui Yi Yung, Letizia Lo Grasso, Abeed Fatima Mohidin, Enzo Acerbi, Jamie Hinks, Thomas Seviour, Enrico Marsili, Federico M. Lauro

Scientific Reports 6: Article number: 1989910.1038/srep19899; published online: 01 28 2016; updated: 04 06 2017

In this Article, the shaded cells and bold numbers in Table 3 have been omitted. The correct Table 3 appears below as Table 1.

As a result, the table legend “*E. coli* contains the ISC and SUF Fe/S assembly system. Treatment: Chemical treatment associated with the DE genes; ID: Gene ID; the shaded cells and bolded numbers are not shown in the table. Genes marked with “*”: Gene promoter-fused GFP assays performed”

should read:

“*E. coli* contains the ISC and SUF Fe/S assembly system. Treatment: Chemical treatment associated with the DE genes; ID: Gene ID; Shaded cells: up regulated DE genes; Bolded number: down regulated DE genes; Genes marked with “*”: Gene promoter-fused GFP assays performed”

In addition, there is an error in the ‘Results and Discussion’ section.

“The cytoplasmic putrescine transporter protein, encoded by *PpotFGHI*, was significantly up regulated following n-butanol, DMA, NMP and T treatment.”

should read:

“The cytoplasmic putrescine transporter protein, encoded by *potFGHI*, was significantly up regulatedfollowing n-butanol, DMA, NMP and T treatment.”

## Figures and Tables

**Table 1:**