# Supplementary material for: Global transcriptomic responses of Escherichia coli K-12 to volatile organic compounds
Source: Sci Rep. 2016 Jan 28;6:19899. doi: 10.1038/srep19899 (PMC4730218; doi:10.1038/srep19899)
Supplement: Supplementary Information [file srep19899-s1.pdf]

Title: Global transcriptomic responses of *Escherichia coli* K-12 to volatile organic compounds.

Authors

Pui Yi Yung[1], Letizia Lo Grasso[1], Abeed Fatima Mohidin[1], Enzo Acerbi[1], Jamie Hinks[1], Thomas Seviour[1], Enrico Marsili[1,2,3], Federico M. Lauro*[1,4]

Affiliations:

1. Singapore Centre on Environmental Life Sciences Engineering (SCELSE). 60 Nanyang Drive, SBS-01N-27, Singapore 637551

2. School of Chemical and Biomedical Engineering, Nanyang Technological University, 62 Nanyang Drive, Singapore 637459

3. School of Biotechnology, Dublin City University, Collins Avenue, Dublin 9, Ireland

4. Asian School of the Environment, Nanyang Technological University, 50 Nanyang Avenue, N2-01C-45, Singapore 639798

Supplementary Methods

Minimal inhibitory concentration assays

MIC assays were performed as follows: *E. coli* K12 strain MG1655 was cultured in 10 mL LB5 broth within a shaking incubator at 150 rpm and at 37 °C for 16 h. The overnight culture was diluted (1:100) in 10 mL MOPs medium (Neidhardt et al, 1974) supplemented with 1.5% glucose and different concentrations of selected VOCs (0%, 0.02%, 0.1%, 0.5%). Optical density readings at 600nm were performed every hour.

Motility assays

Motility assays were performed on *E. coli* in the same growth medium as described above (MOPS medium with 1.5% glucose), supplemented with 0.35% Bacto agar (Difco) and same VOC concentrations as for the RNAseq experiment. Five µL of overnight cultures of *E. coli* were spotted in the center of the solidified agar, plates were then sealed and incubate at 37 °C. Migration diameter (in mm) was measured after 20 h.

Supplementary Tables and Figures:

Table S1: List of DE genes related to cold shock response. Cells in blue: Down regulated DE genes; Cell in yellow: Up regulated DE genes. Asterisk (*): expression of the gene was tested with corresponding promoter clone.

Treatment	ID	Gene	B	CHP	CP	DMA	DMS	NMP	NMS	T	Gene description
b chp cp dma dms nmp nms t	b3556	cspA*	2.92	2.97	2.54	2.39	3.5	3.32	4.09	2.17	RNA chaperone and antiterminator
b chp cp dma dms nmp nms	b1557	cspB*	3.05	4.2	4.34	1.41	3.7	3.07	4.68	0.44	Cold shock protein; Qin prophage
b chp cp dma dms nmp nms	b0880	cspD*	-2.29	-1.14	-2.24	-1.71	-1.3	-2.2	-3.15	-0.39	DNA replication inhibitor, csp homolog
b chp cp dms nmp nms	b1558	cspF	1.33	1.48	2.02	0.81	1.75	1.3	2.92	0.14	Cold shock protein; Qin prophage
b chp cp dma dms nmp nms t	b0990	cspG*	5.1	3.96	4.46	3.46	5	3.91	6.34	1.68	Cold shock protein
b chp cp dms nms	b0989	cspH	1.27	1.99	2.02	1.14	1.88	0.83	3.85	0.14	Cold shock protein
b chp cp dma dms nmp nms t	b1552	cspI*	5.32	7.25	8.54	2.99	4.56	4.55	5.98	2.5	Cold shock protein; Qin prophage
b chp cp dma nmp nms t	b2597	raiA	-2.25	1.19	1.03	-2.19	-0.8	-1.42	-1.38	-1.72	Cold shock protein inhibitor associated with 30S ribosome
b chp cp dma dms nmp nms t	b1544	ydfK	2.83	4.11	5.12	1.54	2.5	2.67	3.88	1.08	Cold shock protein, Qin prophage
b chp cp dms nms	b0991	ymcE*	2.22	1.26	1.41	0.84	1.83	0.94	3.37	-0.45	Cold shock gene
b chp cp dma dms nmp nms	b1375	ynaE	3.09	3.85	5.32	1.52	2.59	2.71	4	1.19	Cold shock protein, Rac prophage
b chp cp dms nmp nms	b1551	ynfN	5.81	6.29	6.95	2.58	3.95	4.42	6.26	1.71	Cold shock-induced protein; Qin prophage

Table S2: List of DE genes related to flagella biogenesis and assembly. Cells in blue: Down regulated DE genes; cells in yellow: Up regulated DE genes.

Treatment	ID	Gene	B	CHP	CP	DMA	DMS	NMP	NMS	T	Description
b chp cp dma dms nmp t	b1072	flgA	-3.22	-4.30	-4.08	-1.22	-1.73	-2.96	-0.06	-3.66	assembly protein for flagellar basal-body periplasmic P ring
b chp cp dma dms nmp t	b1073	flgB	-3.92	-5.88	-4.71	-1.09	-1.81	-3.20	0.14	-4.19	flagellar component of cell-proximal portion of basal-body rod
b chp cp dms nmp t	b1074	flgC	-3.58	-6.69	-4.82	-0.74	-1.60	-3.13	0.49	-4.02	flagellar component of cell-proximal portion of basal-body rod
b chp cp dms nmp t	b1075	flgD	-3.50	-5.75	-4.17	-0.78	-1.46	-3.07	0.61	-3.81	flagellar hook assembly protein
b chp cp dms nmp t	b1076	flgE	-3.32	-5.58	-3.51	-0.65	-1.30	-3.09	0.93	-3.68	flagellar hook protein
b chp cp dms nmp nms t	b1077	flgF	-3.12	-5.74	-3.05	-0.69	-1.01	-2.84	1.22	-3.80	flagellar component of cell-proximal portion of basal-body rod
b chp cp dms nmp nms t	b1078	flgG	-2.90	-5.09	-2.56	-0.57	-1.02	-2.66	1.48	-3.62	flagellar component of cell-distal portion of basal-body rod
b chp cp nmp nms t	b1079	flgH	-2.89	-5.06	-3.78	-0.87	-0.85	-2.75	1.44	-3.67	flagellar protein of basal-body outer-membrane L ring
b chp cp nmp nms t	b1080	flgI	-2.25	-4.53	-3.15	-0.28	-0.71	-2.42	2.09	-3.42	putative flagellar basal body protein
b chp cp nmp nms t	b1081	flgJ	-1.88	-3.46	-2.26	-0.12	-0.47	-2.40	2.42	-3.23	flagellar rod assembly protein and murein hydrolase
b chp nmp nms t	b1082	flgK	-1.51	-1.20	0.19	-0.24	-0.02	-2.96	1.84	-4.02	flagellar hook-filament junction protein 1
b chp nmp nms t	b1083	flgL	-1.28	-1.32	0.39	0.05	-0.22	-2.83	1.55	-3.37	flagellar hook-filament junction protein
b chp nmp t	b1071	flgM	-1.87	-1.31	-0.12	-0.74	-0.78	-2.39	0.56	-2.72	anti-sigma factor for FliA (sigma 28)
b nmp t	b1070	flgN	-1.47	-0.85	0.20	-0.48	-0.52	-1.77	0.73	-2.30	export chaperone for FlgK and Flg
b chp cp dms nmp nms t	b1879	flhA	-2.73	-4.41	-3.49	-0.68	-1.25	-2.16	1.55	-3.45	putative flagellar export pore protein
b chp cp dms nmp nms t	b1880	flhB	-2.15	-4.34	-2.74	-0.44	-1.23	-2.76	1.21	-4.61	flagellin export apparatus, substrate specificity protein
t	b1891	flhC	-0.77	0.16	-0.60	-0.33	-0.43	-0.40	0.46	-1.36	flagellar class II regulon transcriptional activator, with FlhD
b t	b1892	flhD	-1.07	-0.11	-0.84	-0.31	-0.64	-0.70	-0.08	-1.24	flagellar class II regulon transcriptional activator, with FlhC
b chp cp dms nmp nms t	b1878	flhE	-3.15	-2.98	-3.13	-0.77	-1.44	-2.52	1.49	-3.66	proton seal during flagellar secretion
b chp cp dms nmp t	b1922	fliA	-2.65	-3.07	-1.68	-0.67	-1.40	-3.42	0.39	-4.02	RNA polymerase, sigma 28 (sigma F) factor
chp nmp nms t	b1923	fliC	-0.64	-1.41	-0.09	0.17	0.35	-3.32	1.31	-3.33	flagellar filament structural protein (flagellin)
chp nmp nms t	b1924	fliD	-0.89	-1.67	-0.45	0.18	-0.18	-2.90	1.22	-3.50	flagellar filament capping protein
b chp cp dms nmp nms t	b1937	fliE	-2.89	-4.76	-3.50	-0.57	-1.24	-2.60	1.05	-3.82	flagellar basal-body component
b chp cp dms nmp nms t	b1938	fliF	-3.41	-5.99	-6.29	-0.87	-1.07	-3.07	1.24	-4.18	flagellar basal-body MS-ring and collar protein
b chp cp nmp nms t	b1939	fliG	-2.90	-4.26	-4.05	-0.42	-0.87	-2.40	1.82	-3.52	flagellar motor switching and energizing component
b chp cp nmp nms t	b1940	fliH	-3.38	-4.96	-5.40	-0.78	-0.96	-2.78	1.72	-3.87	negative regulator of FliI ATPase activity
b chp cp nmp nms t	b1941	fliI	-2.35	-4.86	-4.48	-0.36	-0.66	-2.25	2.11	-3.58	flagellum-specific ATP synthase (EC:3.6.3.14)
b chp cp nmp nms t	b1942	fliJ	-1.84	-5.67	-3.54	0.06	-0.69	-2.14	2.29	-3.11	flagellar protein; K02413 flagellar FliJ protein
b chp cp nmp nms t	b1943	fliK	-2.07	-4.95	-3.91	-0.30	-0.89	-2.40	2.03	-3.19	flagellar hook-length control protein
b chp cp dms nmp nms t	b1944	fliL	-3.02	-6.82	-4.58	-0.68	-1.43	-2.88	1.15	-4.40	flagellar biosynthesis protein
b chp cp dms nmp nms t	b1945	fliM	-2.76	-5.33	-4.47	-0.48	-1.20	-2.86	1.44	-3.81	flagellar motor switching and energizing component
b chp cp dms nmp nms t	b1946	fliN	-2.94	-4.57	-4.48	-0.58	-1.09	-2.42	1.61	-3.82	flagellar motor switching and energizing component
b chp cp nmp nms t	b1947	fliO	-2.84	-4.75	-3.71	-0.44	-0.85	-2.18	2.22	-4.06	flagellar biosynthesis protein
b chp cp nmp nms t	b1948	fliP	-2.54	-4.52	-3.55	-0.29	-0.51	-2.48	2.42	-3.04	flagellar biosynthesis protein
b chp cp nmp nms t	b1949	fliQ	-2.16	-4.56	-2.79	0.02	-0.63	-1.95	2.78	-2.50	flagellar biosynthesis protein
b chp cp nmp nms t	b1950	fliR	-1.78	-2.48	-2.40	-0.42	-0.71	-1.23	2.74	-2.12	flagellar export pore protein
chp nmp nms t	b1925	fliS	-0.89	-2.19	-0.55	0.09	0.04	-2.14	1.44	-3.14	flagellar protein potentiates polymerization
b chp nmp nms t	b1926	fliT	-1.29	-2.05	-0.56	0.24	0.04	-2.09	1.68	-3.11	putative flagellar synthesis and assembly chaperone
chp cp	b1920	fliY	-0.23	-1.69	-1.85	0.31	-0.72	-0.16	-0.84	-0.07	cystine transporter subunit
b chp cp dms nmp t	b1921	fliZ	-2.03	-3.24	-1.40	-0.32	-1.14	-3.01	0.56	-3.67	RpoS antagonist; putative regulator of FliA activity

Table S3: Transcriptomic profile of ring-hydroxylating oxygenases and transformation genes for aromatic compounds (Diaz2001). Shaded cells: up regulated DE genes; Bolded number: down regulated DE genes.

Ring-hydroxylating oxygenases in *E. coli* K-12

Gene ID	Gene	Gene product	b	chp	cp	dma	dms	nmp	nms	t
b0347	mhpA	3-(3-hydroxyphenyl)propionate hydroxylase	-1.25	0.74	0.25	-2.52	0.04	1.11	0.88	0.78
b2538	hcaE	3-phenylpropionate dioxygenase, large (alpha) subunit	-0.29	-0.57	-0.42	-0.21	-0.46	0.01	-0.47	-0.39
b2539	hcaF	3-phenylpropionate dioxygenase, small (beta) subunit	-0.18	0.93	0.66	0.02	-0.28	0.47	-0.41	-0.41
b2540	hcaC	3-phenylpropionate dioxygenase, ferredoxin subunit	0.59	0.21	0.27	0.01	0.26	0.68	-0.73	-0.05
b2542	hcaD	phenylpropionate dioxygenase, ferredoxin reductase subunit	-0.55	-0.37	-0.39	-0.48	-0.27	-0.23	-0.20	-0.58
b1388	paaA	ring 1,2-phenylacetyl-CoA epoxidase subunit	0.72	-1.29	-0.83	0.22	-0.04	0.53	-0.05	1.51
b1389	paaB	putative ring 1,2-phenylacetyl-CoA epoxidase subunit	-0.37	-0.76	-1.15	0.21	-0.81	0.39	-0.07	0.68
b1390	paaC	ring 1,2-phenylacetyl-CoA epoxidase subunit	-0.27	0.10	-1.76	0.19	-0.11	0.45	0.82	0.90
b1391	paaD	ring 1,2-phenylacetyl-CoA epoxidase subunit	-0.84	0.20	-1.00	-0.04	-0.77	-0.43	-1.33	0.51
b1392	paaE	ring 1,2-phenylacetyl-CoA epoxidase, NAD(P)H oxidoreductase component	-0.04	-1.72	-3.02	0.11	-0.37	-0.68	-0.69	0.37
**Aromatic compounds transformation genes (non-mineralization reactions) in *E. coli* K-12**			**b**	**chp**	**cp**	**dma**	**dms**	**nmp**	**nms**	**t**
b3708	tnaA	tryptophanase/L-cysteine desulfhydrase, PLP-dependent	**-2.61**	**-1.32**	**-3.66**	**-2.64**	**-1.31**	**-2.76**	**-2.92**	2.83
b3843	ubiD	3-octaprenyl-4-hydroxybenzoate decarboxylase	0.30	0.49	0.21	0.31	0.34	0.53	0.79	0.57
b2311	ubiX	3-octaprenyl-4-hydroxybenzoate carboxy-lyase	0.43	-0.01	0.21	0.81	0.45	0.95	1.89	-0.19
b3833	ubiE	2-DMK methyltransferase	0.07	0.74	0.75	0.01	0.21	-0.28	0.56	0.08
b2232	ubiG	bifunctional 3-demethylubiquinone-9 3-methyltransferase	-0.22	-0.18	0.17	-0.03	-0.22	-0.19	-0.31	-0.03
b3835	ubiB (yigR)	regulator of octaprenylphenol hydroxylation	0.42	1.03	0.96	0.33	0.42	-0.27	1.06	0.40
b2907	ubiH (visB)	2-octaprenyl-6-methoxyphenol hydroxylase	-0.15	0.17	0.48	-0.20	0.01	-0.67	-0.21	-0.41
b0662	ubiF (yleB)	2-octaprenyl-3-methyl-6-methoxy-1,4-benzoquinol oxygenase	-0.33	0.28	0.29	-0.30	-0.54	-0.64	-0.54	-0.29
b0596	entA	2,3-dihydro-2,3-dihydroxybenzoate dehydrogenase	0.09	0.11	-0.20	0.83	-0.01	0.11	0.15	1.17
b2260	menE	O-succinylbenzoate-CoA ligase	0.92	-0.06	-0.22	0.78	0.76	0.15	0.88	0.66
b0851	nfsA (nfnA)	nitroreductase A, NADPH-dependent, FMN-dependent	-0.12	0.61	0.04	-0.17	-0.12	-0.75	-1.00	0.32
b0578	nfsB (nfnB)	dihydropteridine reductase	**-1.01**	**-1.13**	**-1.47**	-0.34	-0.90	**-1.22**	**-1.51**	-0.64
b1463	nhoA	N-hydroxyarylamine O-acetyltransferase	-0.78	-0.74	-0.10	-0.75	-0.83	-0.62	-0.95	-0.50
b3801	aslA	putative Ser-type periplasmic non-aryl sulfatase	-0.72	0.03	-0.61	-0.28	-0.31	-0.57	-0.14	-0.91
b1498	ydeN	putative Ser-type periplasmic non-aryl sulfatase	**-2.14**	**-1.58**	**-1.83**	-0.69	**-1.29**	-0.53	**-2.25**	-0.64
b3678	yidJ	sulfatase/phosphatase superfamily protein	0.10	0.06	0.64	-0.16	-0.20	0.31	-0.37	0.06

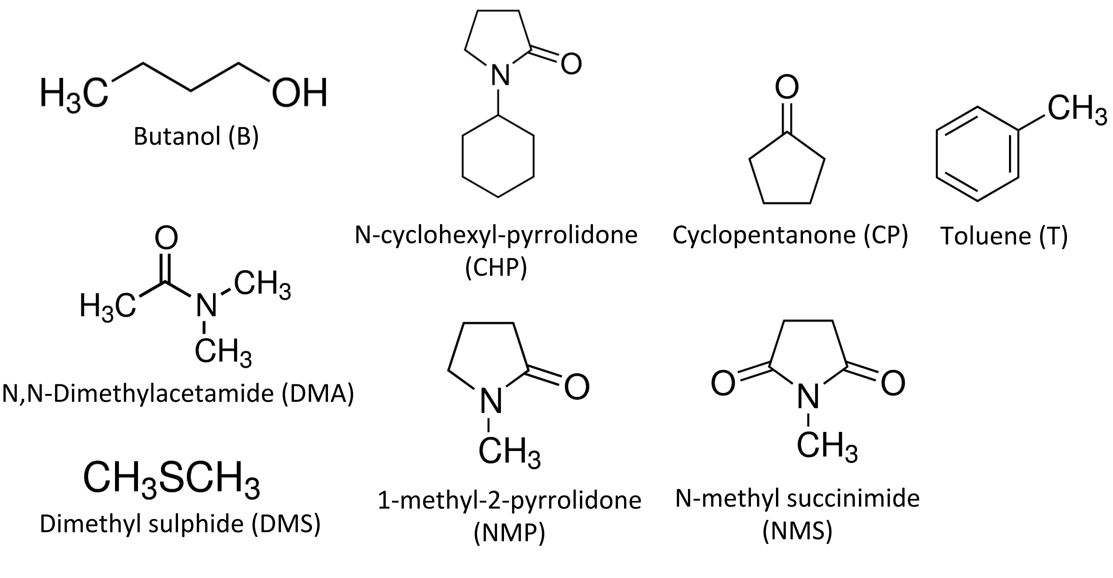

Figure S1: List of compounds tested in the current study.

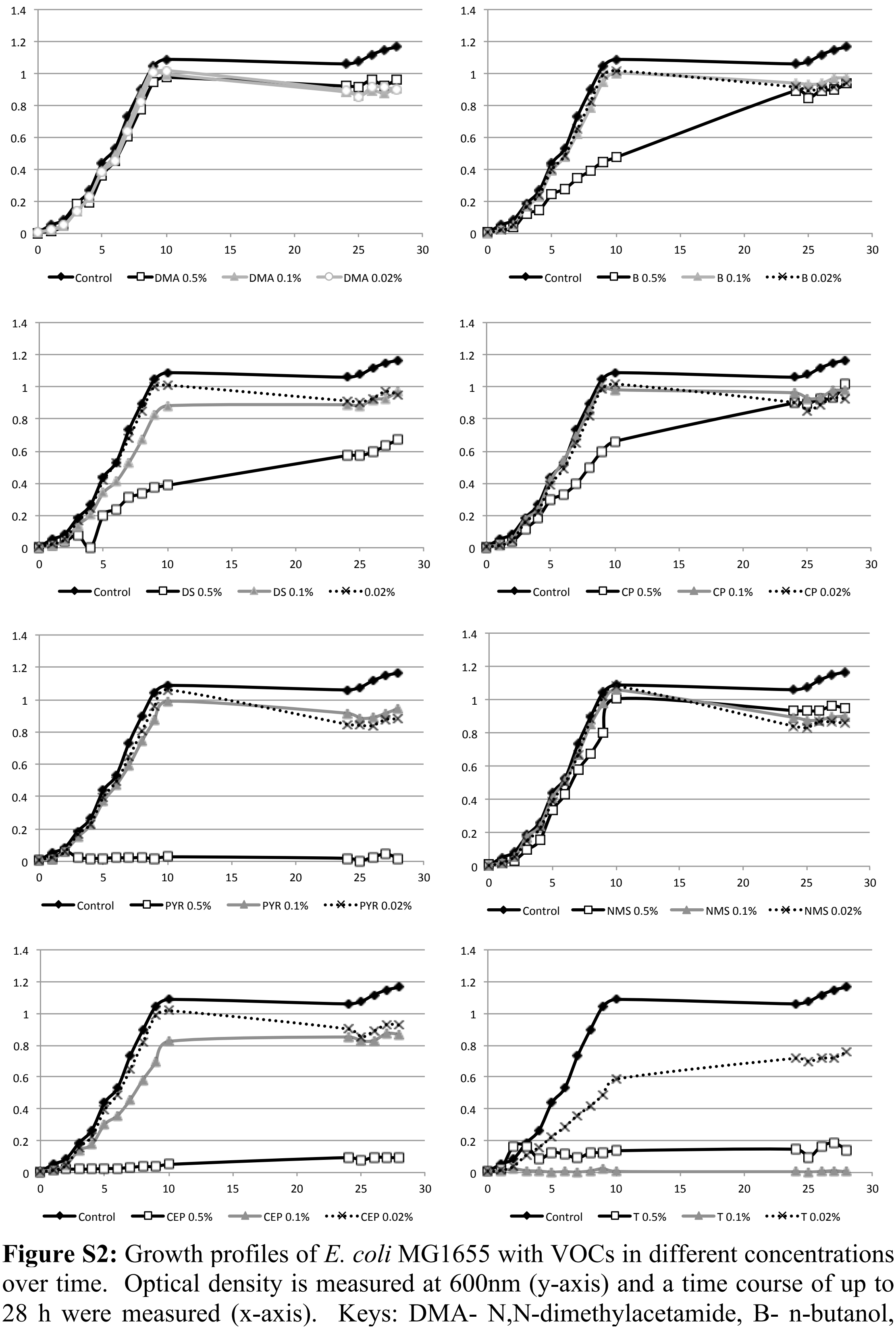

Figure S2: Growth profiles of *E. coli* MG1655 with VOCs in different concentrations over time. Optical density is measured at 600nm (y-axis) and a time course of up to 28 h were measured (x-axis). Keys: DMA- N,N-dimethylacetamide, B- n-butanol, DS- dimethyl sulphide, CP- cyclopentanone, PYR- 2-pyrrolidone-1-methyl, NMS- N-methyl succinimide, CEP- N-cyclohexyl-pyrrolidone, T- Toluene. Symbols (v/v% VOC added): Open square: 0.5%, grey triangle: 0.1%, cross: 0.02%, close diamond: no VOC control.

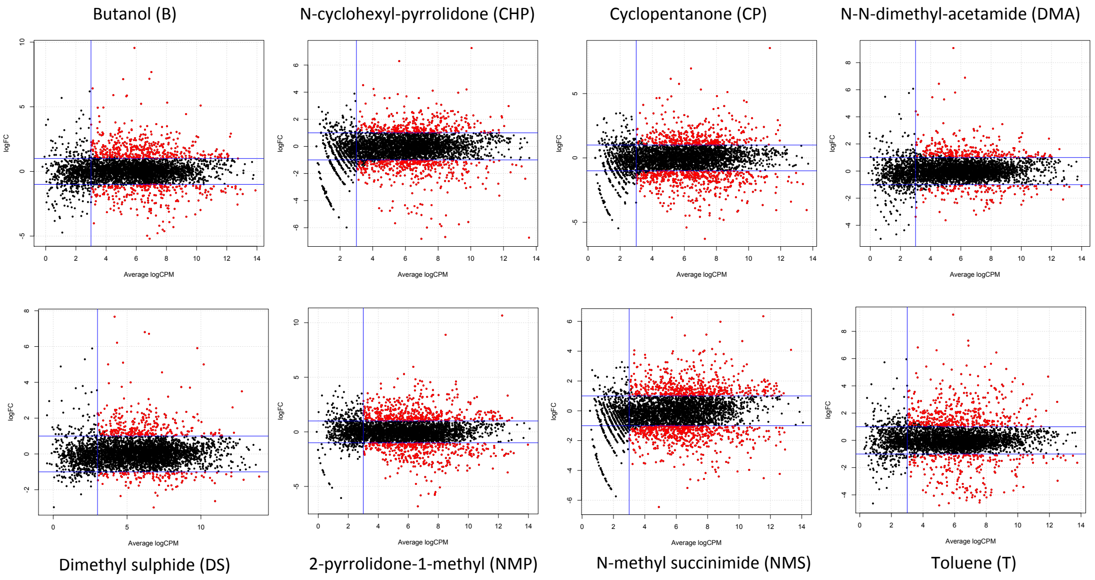

Figure S3: Scatter plot of log-fold change versus average logCPM values. Horizontal blues lines show 2-fold changes (logFC of +/- 1). Vertical blue lines indicate the cut off for low expression genes (average logCPM of more or equal to 3). Red dots highlight the DE genes identified with at least 2-fold changes and a p-value of not more than 0.05 as a cut-off.

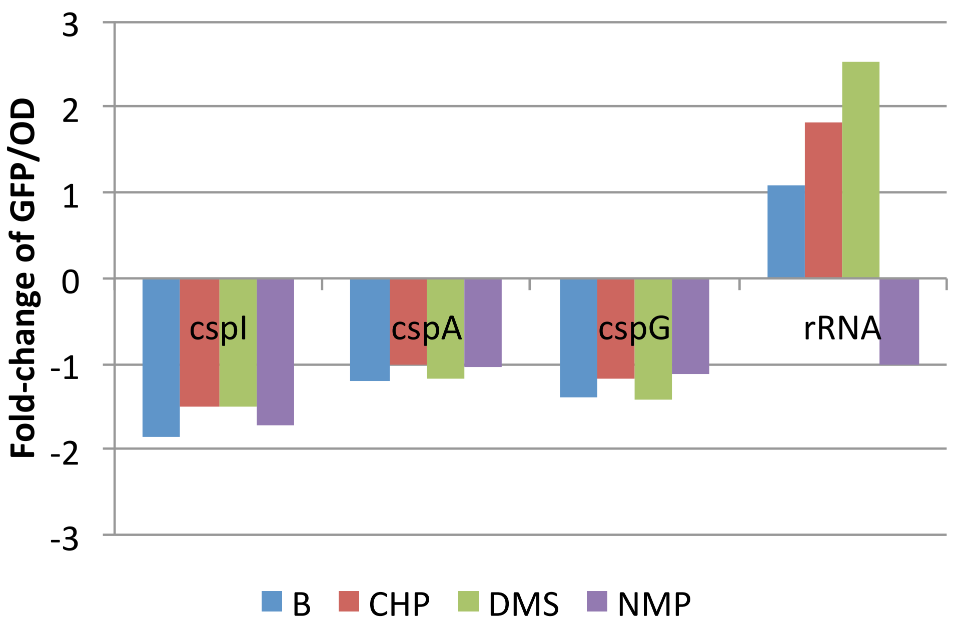

Figure S4: GFP-promoter validation assays of *E. coli* clones containing promoters of selected *csp* genes. Maximum fold-change was obtained from time-series data taken during mid-log phase of cellular growth (approx. 4-6 h from time zero). The fold change of GFP/OD ratio is obtained by comparing with the control treatment without the addition any chemicals. An *E. coli* clone containing promoters of genes encoding for ribosomal RNA protein, was used as comparison.

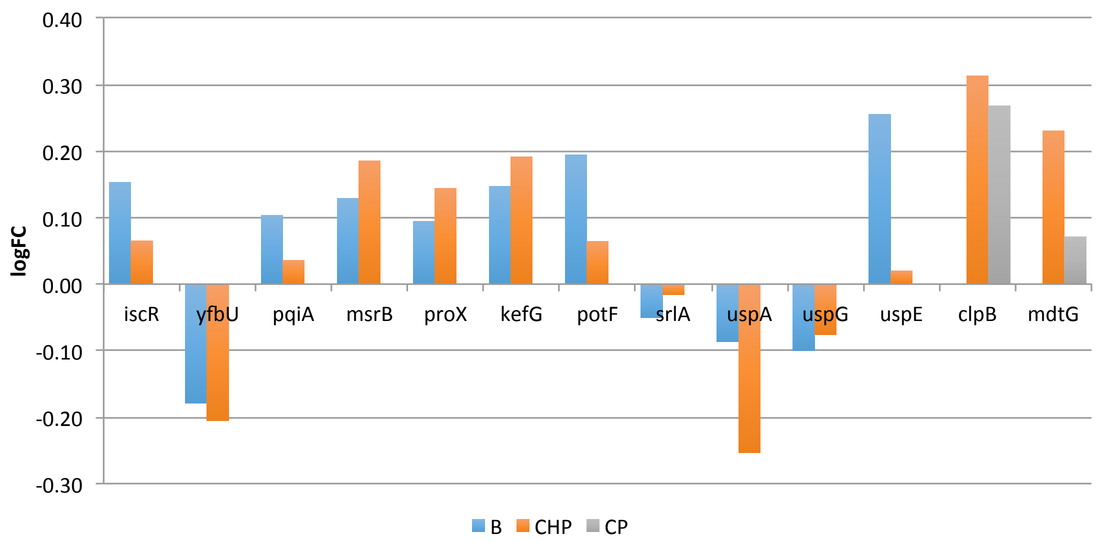

Figure S5: Gene promoter-fused GFP assays performed in *E. coli*. *E. coli* clones with promoter-fused GFP plasmids were used to validate RNAseq results. Cells were grown with the presence of selected VOCs to mid-log phase, followed by GFP measurement. GFP signals were normalized with the O.D. Maximum fold change (compare to the no VOC added control) was recorded. Log fold change of GFP / OD were presented.

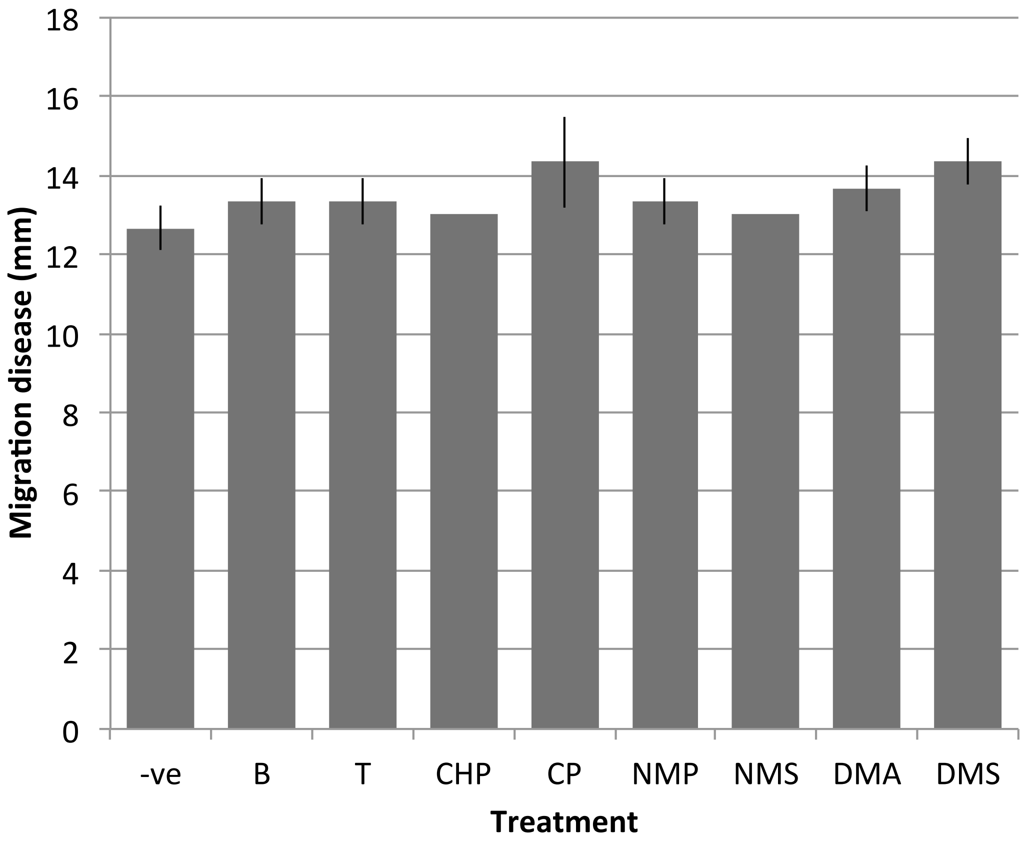

Figure S6: Results from soft-agar motility assay of *E. coli* cells exposed to different chemical treatments. Migration distance at 20 hours is plotted.

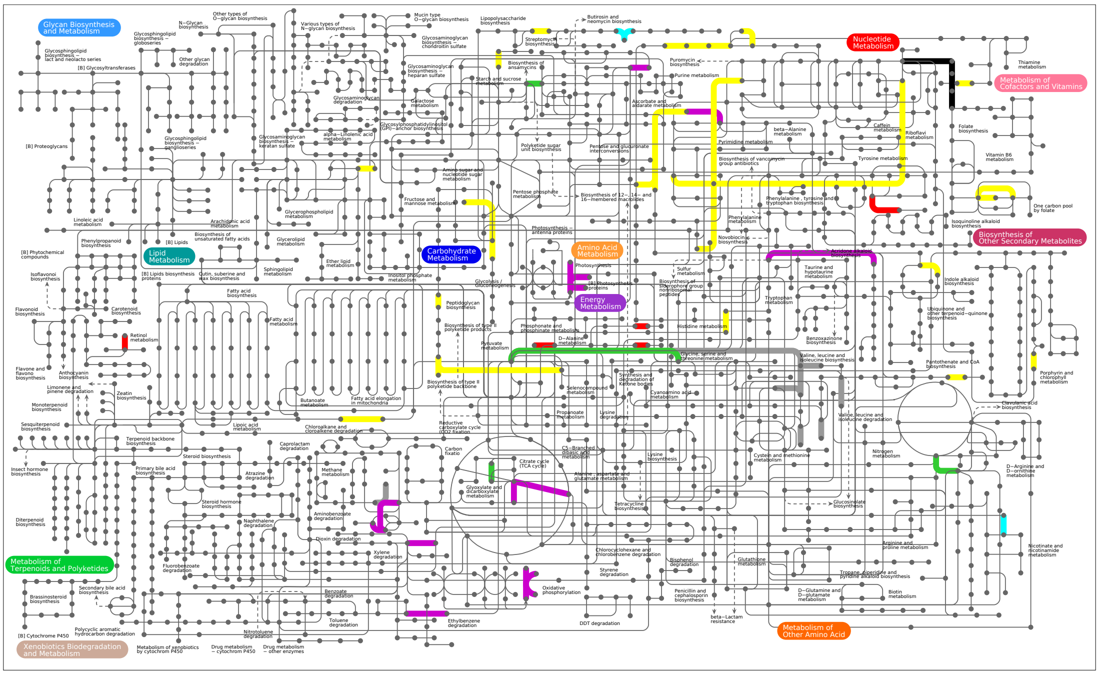

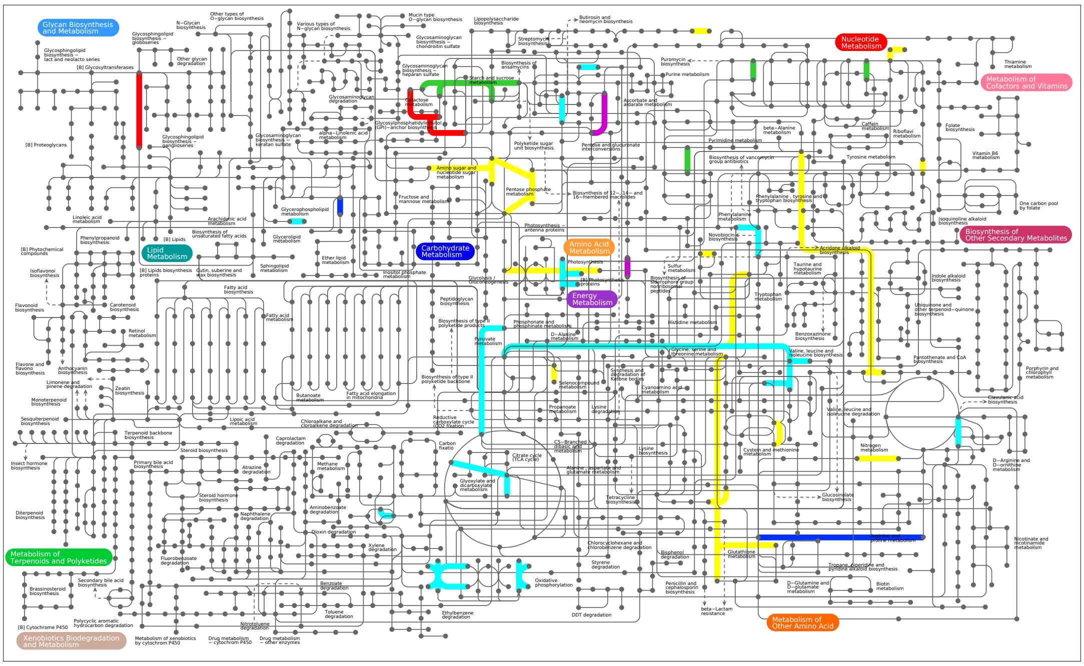

Figure S7: DE genes responded only to single VOC treatment. Colored line represent present of the gene in KEGG metabolic pathway map. Top: Up-regulated genes, Bottom: Down-regulated genes. Key: Red: B; Blue: CHP; Cyan: CP; Black: DMA; Grey: DMS; Green: NMP; Yellow: NMS; Purple: T.
